# Testing for evolutionary change in restoration: A genomic comparison between *ex situ*, native, and commercial seed sources of *Helianthus maximiliani*

**DOI:** 10.1111/eva.13275

**Published:** 2021-07-19

**Authors:** Joseph E. Braasch, Lionel N. Di Santo, Zachary J. Tarble, Jarrad R. Prasifka, Jill A. Hamilton

**Affiliations:** ^1^ Department of Biological Sciences North Dakota State University Fargo ND USA; ^2^ Edward T. Schafer Agricultural Research Center USDA‐ARS Fargo ND USA

**Keywords:** comparative genomics, ecological restoration, evolutionary potential, *ex situ* conservation, genetic bottlenecks, seed provenance, selection

## Abstract

Globally imperiled ecosystems often depend upon collection, propagation, and storage of seed material for use in restoration. However, during the restoration process demographic changes, population bottlenecks, and selection can alter the genetic composition of seed material, with potential impacts for restoration success. The evolutionary outcomes associated with these processes have been demonstrated using theoretical and experimental frameworks, but no study to date has examined their impact on the seed material maintained for conservation and restoration. In this study, we compare genomic variation across seed sources used in conservation and restoration for the perennial prairie plant *Helianthus maximiliani*, a key component of restorations across North American grasslands. We compare individuals sourced from contemporary wild populations, *ex situ* conservation collections, commercially produced restoration material, and two populations selected for agronomic traits. Overall, we observed that *ex situ* and contemporary wild populations exhibited similar genomic composition, while four of five commercial populations and selected lines were differentiated from each other and other seed source populations. Genomic differences across seed sources could not be explained solely by isolation by distance nor directional selection. We did find evidence of sampling effects for *ex situ* collections, which exhibited significantly increased coancestry relative to commercial populations, suggesting increased relatedness. Interestingly, commercially sourced seed appeared to maintain an increased number of rare alleles relative to *ex situ* and wild contemporary seed sources. However, while commercial seed populations were not genetically depauperate, the genomic distance between wild and commercially produced seed suggests differentiation in the genomic composition could impact restoration success. Our results point toward the importance of genetic monitoring of seed sources used for conservation and restoration as they are expected to be influenced by the evolutionary processes that contribute to divergence during the restoration process.

## 1 INTRODUCTION

Restoration aims to mitigate the loss and degradation of native ecosystems by reducing the abundance of non‐native species, increasing biodiversity and habitat connectivity, and re‐establishing native plant communities resilient to change (Benayas et al., 2009; Hobbs & Norton, 1996; Hodgson et al., 2016; Thomson et al., 2009). To achieve these goals, extensive inputs of native seed are required, often in quantities too large to be harvested from local, wild populations (Broadhurst et al., 2008; Merritt & Dixon, 2011; Pedrini et al., 2020). To compensate for these deficits, seeds used in restoration are often produced commercially. However, commercial seed production can lead to the evolution of differences that may impact restoration goals (Dyer et al., 2016; Espeland et al., 2017; Nagel et al., 2019; Pizza et al., 2021; Roundy et al., 1997). Evolution of seed material can occur through bottlenecks and sampling effects following collection, propagation, or cultivation which can lead to reductions in genetic diversity and the loss of locally adapted alleles, impacting fitness and reducing the evolutionary potential of restored populations (Blanquart et al., 2013; Fant et al., 2008; Kawecki & Ebert, 2004; Robichaux et al., 1997; Williams, 2001; Wright, 1938). Combined with selection, which may intentionally or unintentionally lead to genomic and consequent phenotypic change, there is substantial opportunity for evolution during the restoration process (Dyer et al., 2016; Espeland et al., 2017; Nagel et al., 2019). Given the impact different evolutionary processes could have, understanding how these factors interact to influence seed material will have substantial economic and ecological consequences for restoration success (Bischoff et al., 2006, Bucharova et al., 2017, Gerla et al., 2012, Keller et al., 2000, Kimball et al., 2015).

Despite a substantial body of work that has outlined best practices for sampling *ex situ* seed (Griffith et al., 2021; Hoban & Schlarbaum, 2014), selection and sampling effects imposed during collection may also pose a significant challenge to the preservation of genetic variation. *Ex situ* seed collections aim to preserve extant genetic variation to incorporate into restoration or breeding programs in the future (Hamilton, 1994; Li & Pritchard, 2009). Both commercial and *ex situ* seed collections aim to maximize genetic diversity while maintaining locally adaptive genetic variation across space and time (DiSanto & Hamilton, 2020; Griffith et al., 2015). Consequently, genomic comparisons between contemporary wild populations, commercially produced material, and *ex situ* conservation collections provide an ideal means to evaluate the evolution of seed material maintained for conservation and restoration (Robichauxet al., 1997; Schoen & Brown, 1993; Taft et al., 2020). Genomic comparisons of conservation and restoration seed sources with contemporary native populations can be used to infer whether evolutionary challenges inherent to the collection and maintenance of these resources cause them to differ from the wild populations they are intended to match (Pizza et al., 2021).

Sampling effects can generate substantial genomic differences across seed sources with lasting impacts to conservation goals and restoration outcomes (DiSanto & Hamilton, 2020; Diwan et al., 1995; Franco et al., 2005; Hamilton, 1994). The genomic effects of sampling correspond to those found following population bottlenecks, including a reduction in effective population sizes (N_e_) (Leberg, 1992; Wright, 1938), making this a useful metric to compare across populations when quantifying the effects of sampling. In addition, following a bottleneck, rare alleles are more likely to be lost, influencing the distribution of allele frequencies (Excoffier et al., 2009; Maruyama & Fuerst, 1985; Tajima, 1989). Stochastic changes in allele frequencies associated with sampling may also more broadly effect the estimation of inbreeding coefficients (F_is_), linked to inbreeding depression (Cavalli‐Sforza & Bodmer, 1971; García‐Cortés et al., 2010; Husband & Schemske, 1996), or estimates of coancestry (θ), indicative of relatedness among individuals within populations. Importantly, not only are these metrics useful for assessing the magnitude of sampling effects, they are also common proxies for evaluating short‐ and long‐term fitness effects associated with inbreeding depression and increased relatedness among breeding individuals (Angeloni et al., 2011; Caballero & Toro, 2000; Hughes et al., 2008; Keller & Waller, 2002). Thus, these metrics provide valuable comparisons to assess the quality of restoration and conservation seed resources relative to their wild counterparts.

In addition to stochastic processes associated with sampling, directional selection in the agronomic environment can cause evolution during cultivation. This can include selection associated with chemical inputs and fertilizers used to improve yield, reductions in competition, or abiotic stress (Dyer et al., 2016; Espeland et al., 2017). Moreover, individuals with traits promoted by mechanized agricultural harvest, such as reduced shattering, minimum heights, or selected phenology, could evolve in commercially produced material relative to wild populations (Dyer et al., 2016; Nagel et al., 2019). Previous experimental evidence indicates selection can influence the genetic and phenotypic composition of restoration seed (Dyer et al., 2016; Nagel et al., 2019), but no study to date has directly compared the genomic composition of commercially produced seed with native remnant populations in the region of restoration. Genomic signatures of selection can be identified through a variety of statistical analyses developed from the site frequency spectrum (SFS), the distribution of allele frequencies sampled across the genome (Hohenlohe et al., 2010; Nielsen, 2001). Of these metrics, Tajima's D is notable for possessing relatively high statistical power compared to other methods that estimate the strength of selection (Simonsen et al., 1995; Tajima, 1983). If selection occurs during commercial seed production, then we would expect commercial populations to have more negative values of Tajima's D relative to wild populations.

Natural variation in gene flow could also contribute to genetic differentiation among populations, manifesting as isolation by distance (IBD) when genetic differences increase with spatial distance (Slatkin, 1993; Wright, 1943). When present, IBD is expected to produce a positive relationship between genetic differences and the spatial separation between populations and could explain genomic patterns that might otherwise be attributed to selection associated with different seed sources. Consequently, the relationship between geographic and genetic distance can provide a valuable null hypothesis against which alternative evolutionary scenarios can be tested (Bradburd et al., 2016). If genomic differentiation among seed source populations is solely explained by IBD, evolution associated with seed source type has likely not occurred. However, if IBD is absent or is insufficient to explain population differences, other evolutionary factors likely contribute to differentiation across seed source types.

North American grasslands remain one of the most threatened ecosystems globally, with over 98% converted due to anthropogenic development (Comer et al., 2018; Hoekstra et al., 2004; Samson et al., 2004). However, restoration efforts are ongoing to mitigate the loss of native grasslands by planting commercially produced seed mixes (Benayas et al., 2009; Hobbs & Norton, 1996; Thomson et al., 2009). The perennial forb *Helianthus maximiliani* Schrad. (or Maximilian sunflower) is a common constituent of native grassland communities and a frequent component of restoration seed mixes (McKenna et al., 2019; USDA, 2004). *H*. *maximiliani* is distributed across an extensive range of climatic variation spanning a broad latitudinal range from northern Mexico to southern Canada (Kawakami et al., 2014; USDA 2004). Previous genetic studies using microsatellites revealed substantial heterozygosity and low inbreeding rates within populations, consistent with an obligate outcrossing mating system (Kawakami et al., 2014). Quantitative genetic experiments have also found differentiation in traits associated with climatic variation, including freezing tolerance and flowering time (Kawakami et al., 2014; Tetreault et al., 2016). There have also been efforts to breed *H*. *maximiliani* (hereafter selected lines) as a source of seed oil by selecting for increased height, reduced shattering, and increased seed yield (Asselin et al., 2020). Here, we take a genomics approach to evaluate the factors contributing to evolutionary change among *H*. *maximiliani* seed sources to inform both conservation and restoration efforts into the future.

Specifically, we compare contemporary wild populations with seed from *ex situ* collections, seed commercially produced for restoration, and agronomically selected seed to (1) test for differences in genomic composition using ordination and metrics of genetic differentiation, (2) test whether isolation by distance can explain genomic differences among seed source populations, and (3) quantify the impact of sampling and selection across seed source types by comparing genomic summary statistics. With this third objective, we compare statistics that indicate how much genetic variation is maintained across seed sources as a metric of evolutionary potential, including expected heterozygosity (H_e_), inbreeding coefficients (F_is_), and linkage disequilibrium effective population size (LD‐N_e_). We also estimate and compare parameters that can be used to evaluate whether sampling effects or the impact of selection contribute to genomic differences across seed sources. This includes F_is_ and LD‐N_e_, in addition to coancestry (θ), and Tajima's D. Overall, this study serves as an important test of recent hypotheses that identify the role evolutionary processes can play throughout the collection, propagation, and implementation stages of conservation and restoration. Our results provide valuable guidance for the future collection and deployment of seed for restoration while identifying new avenues of research that can address the evolutionary consequences of seed collection and cultivation.

## 2 METHODS

### 2.1 Population sampling

To assess the impact of demographic variation and unintentional selection on the evolution of seed material used in restoration, we compared the genomic composition of contemporary wild populations to seed from three distinct sources: seed collected and/or cultivated by commercial suppliers for restoration, seed preserved in *ex situ* collections, and lines selected for agronomic traits (hereafter selected lines) to assess the impact demographic variation and unintentional selection may have had on the evolution of seed material used in restoration.

**FIGURE 1:**
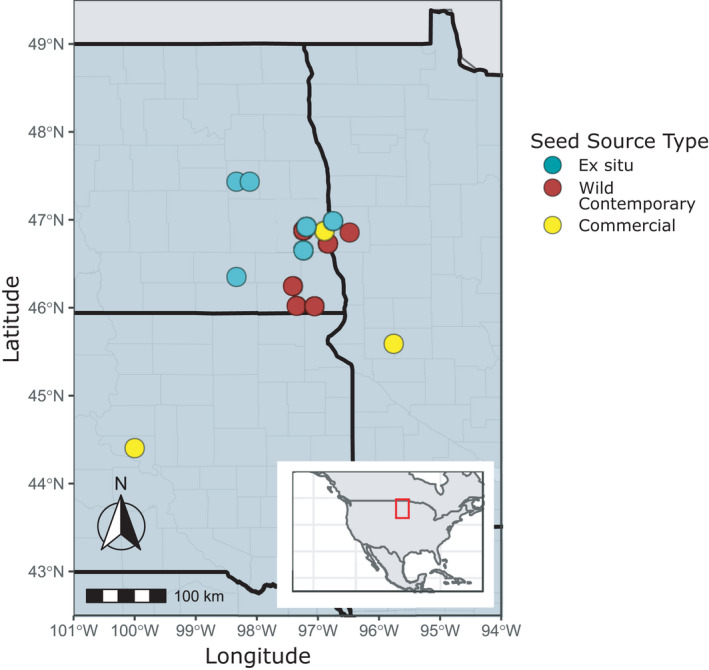
Sampling locations of *Helianthus maximiliani* seed across the northern United States. Location data were available for all native remnant and *ex situ* seed sources and three of the five commercial seed sources used in this study. Location data for the remaining commercially produced seed were not available

During the summer of 2016, tissue was sampled across six naturally formed wild contemporary populations of *H*. *maximiliani*, separated by at least 15 km, across North Dakota and Minnesota (Figure 1). Collection sites reflect remnant *H*. *maximiliani* populations maintained in the region, despite extensive land conversion, as native prairie fragments. Leaf tissue was sampled by randomly collecting leaves from 20 individuals per population along a 100 m transect (Table 1). Following collection, leaves were preserved in silica gel prior to DNA extraction. Four commercial restoration seed suppliers within North and South Dakota provided five seed populations of *H*. *maximiliani* for use in this study. Commercial seed was produced either through direct harvest from the wild or by cultivating local genotypes (Table 1). All commercial seeds were harvested between 2016 and 2019.

**TABLE 1:** Geographic location, sample size, and year of harvest for *Helianthus maximiliani* seed sources

Seed source	Population ID	*n*	State collected from	Latitude	Longitude	Cultivated	Year of harvest
*Ex situ*	ES‐A	20	ND	47.4333	−98.3333	N	1991
	ES‐B	20	ND	46.9167	−97.1833	N	1991
	ES‐C	19	ND	47.4333	−98.1167	N	1991
	ES‐D	19	ND	46.3500	−98.3333	N	1991
	ES‐E	20	MN	46.9833	−96.7500	N	1995
	ES‐F	20	ND	46.6500	−97.2333	N	1995
Wild Contemporary	W‐1	13	ND	46.8758	−97.2321	N	2016
	W‐2	20	MN	46.8554	−96.4814	N	2016
	W‐3	13	ND	46.7278	−96.8339	N	2016
	W‐4	20	ND	46.2459	−97.4060	N	2016
	W‐5	20	ND	46.0216	−97.3462	N	2016
	W‐6	20	ND	46.0177	−97.0537	N	2016
Commercial	C‐1	20	MN	45.5895	−95.7600	N	2017
	C‐2	20	ND	46.8697	−96.8903	Y	2018
	C‐3	20	SD	44.4031	−99.9997	Y	2016
	C‐4	20	SD	‐	‐	Y	2016
	C‐5	19	ND	‐	‐	N	2017
Selected lines	S‐1	20	KS	‐	‐	Y	2014
	S‐2	20	KS	‐	‐	Y	2014

Note

n: number of individuals in each population retained for genetic analysis.

State Collected From: KS, Kansas; MN, Minnesota; ND, North Dakota; and SD, South Dakota.

Cultivated: N, seed collected from naturally growing stands and Y, grown in an agroecosystem for at least one generation prior to seed harvest.

*Ex situ* seed populations included in this study were sourced from the USDA National Genetic Resources Program (https://www.ars‐grin.gov/). These bulk seed collections, designated by local provenance, were collected in North Dakota in September of 1991 (4 collections) and 1995 (2 collections). *Ex situ* seeds were bulk‐harvested by clipping mature seed heads, following which seed heads were dried and cleaned prior to storage in a cold room at 4℃ with 25% humidity.

Selected lines represent germplasm developed as part of a domestication program to improve the agronomic value of perennial grassland species. Breeding populations of *H*. *maximiliani* were originally founded with 10 plants from each of 96 wild populations (960 plants total) harvested in Kansas, US. Each line was bred for five generations selecting for increased yield per stem, yield per seed head, and seed size by pooling pollen from the twenty best performing families in each generation and using pooled pollen to fertilize plants from the same twenty families. Seeds were harvested in 2014 and stored at 4℃.

To obtain leaf tissue for genomic analysis, in 2018 we grew seeds sourced from commercial, *ex situ*, and selected lines indoors and under growth chamber conditions in Fargo, ND. Seeds were germinated in bulk following the protocol by Seiler (2010). Achenes were surface‐sterilized and soaked for 15 min in a 2% solution of 5.25% sodium hypochlorite in distilled water with a single drop of wetting agent (Tween 20, Sigma‐Aldrich, Inc. St. Louis, MO, USA). Achenes were then rinsed and scarified with a razor blade, cutting through the hull and tip of cotyledons before soaking in a 100 PPM solution of gibberellic acid (Sigma‐Aldrich, Inc.) for 60 min. Following this, achenes were placed onto filter paper in Petri plates, sealed with parafilm, and stored overnight in the dark at 21℃. Seeds (embryos) were gently removed from hulls, returned to Petri plates and examined daily for germination. Seeds with a visible radicle were planted into a moistened peat pellet (Jiffy Peat Pellet, Plantation Products, Norton, MA, USA) and grown at 21℃ under artificial lights (fluorescent T12 bulbs) until they produced between 4 and 8 true leaves. Leaf tissue samples were collected from 20 randomly selected individuals from each population or seed collection (20 individuals × 13 sources = 260 total individuals) and stored in silica gel prior to DNA extraction.

### 2.2 DNA sequencing and genotyping

We extracted DNA from ~10 mg of dried leaf tissue using a modified Macherey‐Nagel NucleoSpin Plant 2 extraction kit that included additional ethanol washes to ensure removal of secondary plant compounds. DNA concentration was verified using the Quant‐iT™ PicoGreen^®^ dsDNA kit (Life Technologies, Grand Island, NY) after submission to the University of Wisconsin‐Madison Biotechnology Center for sequencing. Genomic libraries were prepared as in Elshire et al. (2011) with minimal modification. In short, 50 ng of DNA was digested using the 5‐bp cutter ApeKI (New England Biolabs, Ipswich, MA) after which barcoded adapters were added by ligation with T4 ligase (New England Biolabs, Ipswich, MA) for Illumina sequencing. The 96 adapter‐ligated samples were pooled and amplified to provide library quantities appropriate for sequencing, and adapter dimers were removed by SPRI bead purification. Fragment length and quantity of DNA were measured using the Agilent Bioanalyzer High Sensitivity Chip (Agilent Technologies, Inc., Santa Clara, CA) and Qubit^®^ dsDNA HS Assay Kit (Life Technologies, Grand Island, NY), respectively. Libraries were standardized to 2 nM and were sequenced using single read, 100 bp sequencing and HiSeq SBS Kit v4 (50 Cycle) (Illumina Inc.) on an Illumina HiSeq 2500. Cluster generation was performed using HiSeq SR Cluster Kit v3 cBot kits (Illumina Inc., San Diego, CA, USA).

Sequence files were demultiplexed using *ipyrad* version 0.9.12 (Eaton 2014) allowing for zero mismatches in the barcode region. Following demultiplexing, single nucleotide polymorphisms (SNPs) were called across populations and seed sources using the dDocent v2.7.8 pipeline (Puritz et al., 2014, 2014,2014, 2014). In the first step of the pipeline, reads were trimmed using the program TRIMMOMATIC (Bolger et al., 2014), including the removal of low‐quality bases and Illumina adapters. Following read trimming, the pipeline aligned reads to the *Helianthus annuus* v1.0 genome using BWA (Li & Durbin 2009). Sequence alignment was performed using the software's default parameters (a match score of 1, mismatch score of 4, and gap score of 6). Finally, as a last step, dDocent called SNPs using the software FREEBAYES (Garrison & Marth 2012) that produced a VCF file with 4,735,557 total SNPs. Downstream SNP filtering of the VCF file first removed missing loci variants with conditional genotype quality (GQ) <20 and genotype depth <3. Then, loci with Phred scores (QUAL) ≤30, allele counts <3, minor allele frequencies <0.05, call rates across all individuals <0.9, mean depth across samples >154 (based on the equation from Li, 2014), and linkage scores >0.5 within a 10 kb window were removed. Following downstream filtering, 12,943 polymorphic loci were kept and used for subsequent analyses. Individuals with more than 30% missing genotypes were removed from the analysis. In total, 14 individuals from wild contemporary populations, two individuals from *ex situ* collections, and one individual from a commercial supplier were discarded, leaving a total of 363 genotyped individuals for inclusion in subsequent analysis (Table 1).

### 2.3 Population structure and genetic differences between seed sources

To test for the effects of seed source on the genetic structure among *H*. *maximilani* populations, we used principal component analysis (PCA) and discriminant analysis of principal components (DAPC) to partition the genetic variation observed in our sampling. Pairing these methods provides valuable insight as it allows comparison of a method agnostic to *a priori* expectations for population structure (PCA) to one which attempts to best depict differences between populations (DAPC). Additionally, while PCA allows for the visualization of individual axes which explain decreasing amounts of the total genomic variation, DAPC can combine and display variation across multiple axes of variation simultaneously. DAPC accomplishes this by first partitioning genetic variance using PCA and then using discriminant analysis to maximize interpopulation variation while minimizing intrapopulation variation. This allows DAPC to identify the axes of variation that simultaneously maximize between group differences and minimize within group differences (Jombart et al., 2010). Thus, DAPC will isolate and incorporate only those axes that contribute to differences between our populations, while PCA depicts population groupings onto major axes of variation.

We performed principal component analysis (PCA) on the matrix of SNPs used for all individuals in the study. Missing data (2.5% of all loci) were substituted with the mean allele frequencies at each locus. We calculated the total variation explained by each axis by dividing the eigenvalue of each PCA and the total sum of all eigenvalues. PCA was performed with the *dudi*.*pca* function within the ADEGENET package (Jombart 2008; Jombart & Ahmed 2011). We then plotted individuals along the first two PCA axes using *s*.*class* function in the package ADEGRAPHICS (Siberchicot et al., 2017).

We first applied DAPC to the entire SNP dataset and then to a subset of the data including only individuals from wild contemporary and *ex situ* populations. Cross‐validation identified the optimal number of PC axes (175 and 108, respectively) necessary to describe among population differences for analysis of all populations and for the *ex situ* and wild contemporary population comparison alone. We then retained 18 and 11 discriminant functions for depicting between group differences for all seed sources and *ex situ and* wild contemporary analysis, respectively. All DAPC analyses were performed using the R package ADEGENET (Jombart 2008).

### 2.4 Isolation by distance in seed collections

Genomic differences across populations can arise from the independent evolution of populations connected by limited gene flow giving rise to isolation by distance (IBD). Patterns of neutral evolution produced by IBD could create genomic differences that are erroneously attributed to the effects of selection or sampling. For this reason, we tested for any correlation between F_st_ calculated between two populations and the spatial distance between them. Testing for IBD was also necessary due to the uneven spatial distribution of populations from different seed sources. If we identify a positive signal of IBD, then genomic differences could be due to the spatial arrangement of sampling, rather than any evolved differences. Therefore, it is necessary to test for IBD to confidently attribute genomic differences to environmental or sampling effects associated with different seed source types in our later analyses.

Pairwise genetic differences between populations were calculated as F_st_ using the Weir and Cockerham's method which is unbiased with regard to differences in sample sizes (Weir & Cockerham 1984; Willing et al., 2012). Unlike DAPC, pairwise F_st_ allows us to quantify the total genetic differences between population pairs. Importantly, we can compare the magnitude of genetic differences for populations of the same seed source type (e.g., two wild contemporary populations) to differences calculated between populations of different seed source types (e.g., wild contemporary population versus commercial population). Therefore, before testing for IBD, we confirmed that estimates of pairwise F_st_ indicated the presence of genomic differences between seed sources and were similar to patterns of differentiation observed with DAPC and PCA. If evolved differences between populations have developed due to conditions associated with seed source type, we expect inter‐source F_st_ to be larger than intra‐source F_st_. To test for differences in inter‐ and intra‐source Fst, we used a Wilcoxon rank sum test implemented with the function “wilcox.test” in R.

To test for effects of IBD and seed source types on pairwise F_st_, we first calculated the geographic distance between seed collections. Exact geographic location data were available for all wild contemporary and *ex situ* populations. Locations for commercial populations C‐1, C‐2, and C‐3 were estimated as approximate locations based on descriptions of the counties, cities, reserves, and geographic features associated with the provenance for each collection. Provenance data were not available for the remaining two commercial populations (C‐4 and C‐5) and selected lines (S‐1 and S‐2), and therefore, these populations were not included in the analysis. Geographic distances were calculated using the haversine formula, which accounts for the curvature of the earth (Robusto 1957), and then square root transformed to improve model fits. Distance measurements were made using with the R package GEODIST.

The relationship between F_st_, distance, and seed source types used to calculate F_st_ was evaluated using model selection with a series of linear mixed models. In these models, F_st_ was expressed as function of spatial distance (fixed effect) and a factorial variable coded for the different pairwise seed source comparisons with random slopes and intercepts. The variable for seed source comparisons required six levels in total, three for each of the intra‐source comparisons (wild to wild, *ex situ* to *ex situ*, and commercial to commercial) and three for each of the inter‐source comparisons (wild to *ex situ*, wild to commercial, and *ex situ* to commercial). We compared the full model which included both spatial distance and seed sources to two reduced models each of which included only one of the terms. A likelihood‐ratio test, implemented with *lrtest* function in the package LMTEST (Zeileis & Hothorn 2002), was used to identify significant differences between the full and reduced models. When the full and reduced models were significantly different, we chose the model with the greatest log‐likelihood value as the model with the best fit.

### 2.5 Signatures of sampling and selection

To ascertain the importance of sampling effect and selection in contributing to differences among seed sources, we calculated expected heterozygosity (H_e_), inbreeding coefficients (F_is_), linkage disequilibrium effective population size (LD‐N_e_), and coancestry coefficients (θ). Expected heterozygosity (H_e_) and inbreeding coefficients (F_is_) were calculated individually for each SNP using the R package ADEGENET v2.1.0 (Jombart 2008; Jombart & Ahmed 2011). To estimate LD‐N_e_, we followed the method of Braasch et al. (2019) which, rather than producing a single, genome‐wide value, uses the mean from a distribution of LD‐N_e_ estimated using multiple subsets of the data. This method reduces the likelihood of violating the assumption of no physical linkage among loci in organisms without assembled genomes by repeatedly sampling a smaller subset of loci. To produce a distribution of LD‐N_e_ estimates, we created 5,000 sets of 500 loci and estimated LD‐N_e_ for each using the function ldNe in the package StrataG (Archer et al., 2017) following methods from Waples et al. (2016). Estimates of relatedness (θ) were made using the R package COANCESTRY (Wang 2011) with 2,000 bootstrap iterations to calculate 95% confidence intervals for each population.

We compared H_e_ and F_is_ across seed source types using linear mixed models with seed source type as a fixed effect and population as a random effect. The significance of individual terms and post hoc tests were performed with the R package LMERTEST (Kuznetsova et al., 2017). Differences between LD‐N_e_ and θ among wild contemporary, *ex situ*, and commercial seed sources were compared using linear models implemented with the *lm* function. Selected populations were not included in linear models due to lack of replication (*n* = 2).

To test for signatures of selection or bottlenecks across seed source types, we calculated Tajima's D for each population (Tajima 1989). Positive estimates of Tajima's D are indicative of high heterozygosity or a scarcity of rare alleles, which could be caused by balancing selection or demographic bottlenecks, as well as sampling effects. Conversely, recent population expansion or directional selection should result in negative values of D arising from an excess of rare alleles. Whole‐genome estimates of Tajima's D with accompanying p‐values were produced with the “tajima.test” function in the R package PEGAS and compared across seed source types with an analysis of variance (ANOVA) and Tukey post hoc test (Paradis 2010).

**FIGURE 2:**
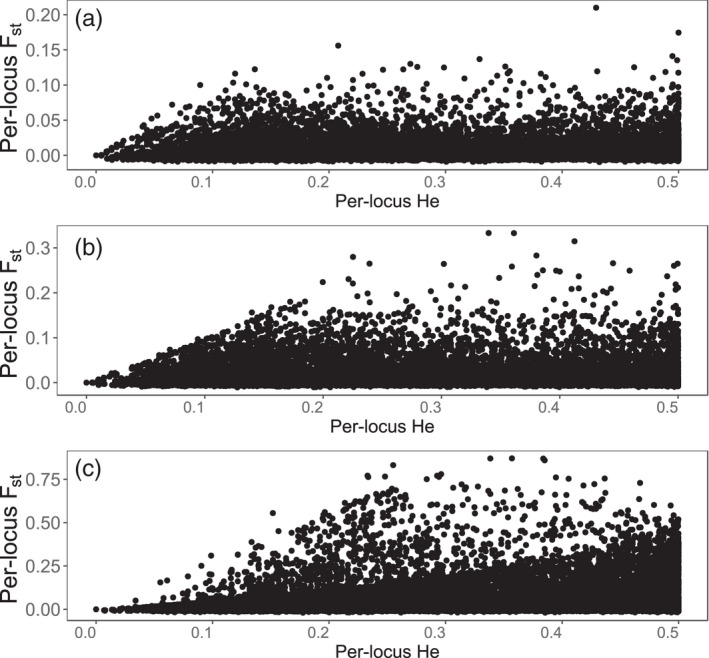
The per‐locus relationship between F_st_ and H_e_ for (a) wild and *ex situ* populations, (b) wild and commercial populations, and (c) wild and selected populations. In all comparisons, there are no loci with low H_e_ and moderate to high F_st_, which matches patterns from simulation studies in which the effects of selection and neutral evolution are equivalent in magnitude

We also note here that plotting per‐locus F_st_ as a function of H_e_ revealed that the data were depauperate in low H_e_, high F_st_ loci (Figure 2). This pattern has been found in other work and matches the expected relationship for these variables when drift and selection contribute similarly to evolutionary differentiation (Narum & Hess 2011). When drift and selection similarly impact the genome, accounting for neutral differentiation in outlier analysis could increase type II error, while failing to account for it would increase type I error. As a result, outlier analyses are not expected to yield reliable results and were therefore not considered in this manuscript.

To visualize the frequency of rare alleles and overall genetic diversity across seed source types, we constructed a folded site frequency spectrum (SFS) for each seed source, with the exception of the selected lines. SFSs were estimated from the filtered SNP dataset (12,943 variants, 363 individuals) using the set of R functions available at https://github.com/shenglin‐liu/vcf2sfs. Individuals from populations classified as the same seed source type (Table 1) were pooled together to generate seed source‐specific allele frequency profiles.

## 3 RESULTS

### 3.1 Population structure and genetic differences between seed sources

**FIGURE 3:**
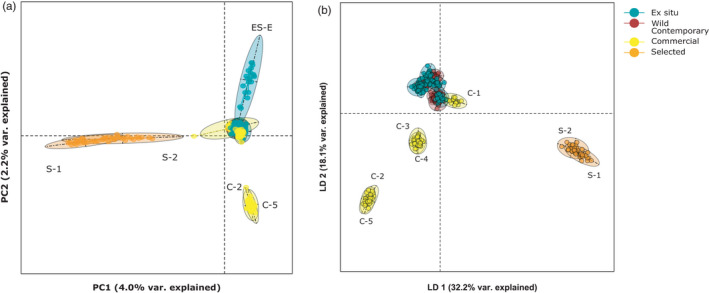
Genomic variation of *Helianthus maximiliani* partitioned by (a) principal component analysis (PCA) and (b) discriminant analysis of principal components. Both analyses were conducted on the full SNP dataset for all *ex situ*, wild contemporary, commercial, and selected populations. In panel (a), wild contemporary individuals are behind the dense cluster of *ex situ* and commercial individuals just to the right of the origin. Missing data (2.5% of all observations) in the PCA were replaced with the mean allele frequency value. Different seed source types are depicted as different colors (turquoise: *ex situ*; red: wild contemporary; yellow: commercial; and orange: selected)

In total, 363 individuals and 12,943 SNP loci passed our filtering requirements. PCA of the entire dataset required 110 axes to explain over 50% of the total genetic variation across all seed source types. A total of 4.0% of the total genetic variation was explained by the first principal component axis, which differentiated the two selected populations from all other seed sources (Figure 3A). The second axis explained 2.2% of the total genetic variation and separated *ex situ* population ES‐E and commercial populations C‐2 and C‐5 on either end of the axis and from all remaining populations at the center.

When genetic variation was partitioned for all seed sources using DAPC, the first two axes explained 32.2% and 18.1% of genetic variation, respectively (Figure 3B). These values are considerably greater than PCA axes because DAPC incorporates and depicts the relationships across multiple axes of variation simultaneously. DAPC, which also attempts to maximize differences between predefined groups, split populations of *H*. *maximiliani* into four distinct groups. All wild contemporary, all *ex situ*, and commercial population C‐1 from Minnesota formed one group together. The remaining four commercial populations were split into two clusters according to the state they were sourced from. Commercial populations from North Dakota, C‐2 and C‐5, grouped together, as did the commercial populations from South Dakota, populations C‐3 and C‐4. Selected populations formed their own unique cluster. The first DAPC axis separated selected populations from all others. The second DAPC axis splits the two clusters of commercial populations from the cluster of wild contemporary populations, *ex situ* populations, and population C‐1.

A DAPC including only *ex situ* and wild contemporary seed explained 40.1% of the total variation in this subset of the data (Figure S1). The first and second ordination axes explained 24.1% and 16.0% of genomic differences, respectively. Populations did not split according to seed source type, although three *ex situ* populations (ES‐A, ES‐C, and ES‐E) were separated from most other populations along axis 2.

### 3.2 Isolation by distance in seed collections

Pairwise F_st_ ranged from −0.001, between commercial populations C‐2 and C‐5, to 0.238 between *ex situ* populations ES‐E and selected population S‐1 (Figure S2). Negative F_st_ likely arises due to similarity between populations and the infrequent presence of polymorphic loci at otherwise monomorphic sites (Roesti et al., 2012). Pairwise F_st_ mirrored patterns observed in PCA. The largest values of F_st_ observed were between selected and nonselected populations. Additionally, F_st_ values calculated with population ES‐E were larger than F_st_ calculated using any other *ex situ* collections. Across all pairwise F_st_, inter‐seed source comparisons were significantly greater than intra‐seed source comparisons (Wilcoxon signed rank test: W = 3,845, *p* < 0.001) (Figure S3). The linear mixed model using pairwise F_st_ as the dependent variable and seed source comparison as the only independent variable was significantly better than the full model which included seed source comparison and geographic distance (Table 2). The model using only distance as an independent variable was not significantly different from the full model.

**TABLE 2:** The effect of isolation by distance and seed source on pairwise F_st_

Model	Log‐likelihood	Df	*p*‐value
Distance + seed source comparison	223.75	‐	‐
Distance	223.49	1	0.42
Seed source comparison	228.98	1	**0.001**

Note

Reduced models were compared to the full model using the likelihood‐ratio test. *p*‐values in bold indicate significant differences between the reduced and full model.

### 3.3 Patterns of genetic diversity and relatedness

**FIGURE 4:**
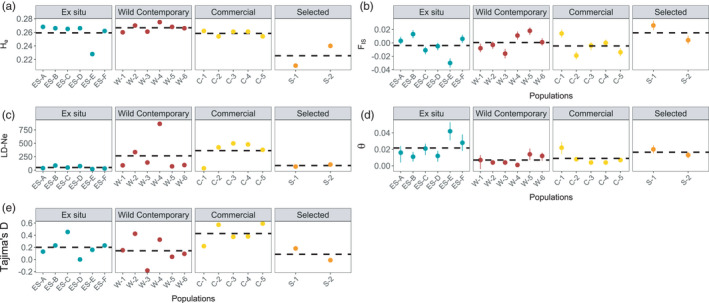
Estimates of (a) expected heterozygosity (H_e_), (b) inbreeding coefficients (F_is_), (c) linkage disequilibrium effective population size (LD‐N_e_), (d) coancestry coefficients (θ), and (e) genome‐wide Tajima's D for commercially produced, *ex situ*, native remnant, commercial, and experimentally selected *Helianthus maximiliani* seed sources. The dashed line depicts the mean estimate of each statistic for each seed source. Points depict population means for panels a and b, and error bars depict standard errors. Points depict absolute estimates for panels c–e. Error bars for LD‐N_e_ and θ are bootstrapped 95% confidence intervals estimated with 2000 replicates

Average H_e_ for all *H*. *maximiliani* populations ranged between 0.211 ± 0.002 SE and 0.275 ± 0.001 SE (Figure 4A). H_e_ was similar across wild contemporary, *ex situ*, and commercial populations, all of which were significantly greater than H_e_ in selected lines (full model: F_3,15_ = 6.9, *p* = 0.004) (post hoc tests: wild contemporary‐selected: *p *< 0.001, *ex situ*‐selected: *p* = 0.001, commercial‐selected: *p* = 0.002) (Figure 4A). F_is_ estimates ranged from −0.019 ± 0.001 SE to 0.018 ± 0.002 SE, and there were no significant differences across seed source types (F_3,15_ = 1.1, *p* = 0.363) (Figure 4B).

Wild contemporary and commercial seed populations spanned a wide range of LD‐N_e_ estimates in comparison with *ex situ* and selected populations (Figure 4C). Despite these trends, we did not observe significant differences in LD‐N_e_ between seed source types (F_2,14_ = 3.3, *p* = 0.069). Patterns of coancestry across seed source types mirrored those for LD‐N_e_, and the linear model comparing the effect of seed source was significant (F_2,14_ = 3.6, *p* = 0.037). Although wild contemporary and commercial populations had lower θ (range 0.001–0.022) than *ex situ* and selected populations (0.011–0.042), post hoc comparison revealed only e*x situ* and commercial populations were significantly different (Figure 4D).

**FIGURE 5:**
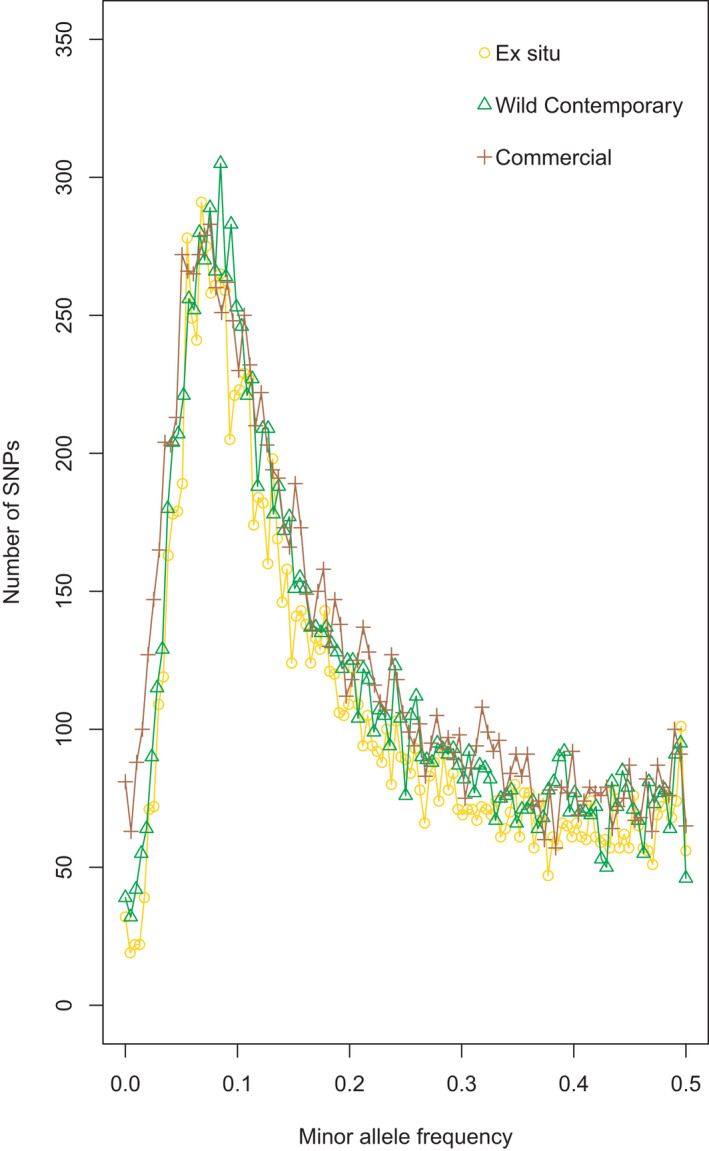
The site frequency spectrum for wild contemporary, *ex situ*, and commercial seed collections shows a greater number of low frequency alleles in commercial populations

Genome‐wide estimates of Tajima's D were not significantly different from neutral expectations for any population, including selected lines (Table S1). Nonetheless, Tajima's D estimated for commercial populations was significantly greater than those for wild contemporary populations (analysis of variance: F_2,14_ = 3.82, *p* = 0.048; Tukey post hoc test wild and commercial: *p* = 0.047) (Figure 4E) suggesting differences in the site frequency spectrum among populations for these two seed sources. The shape of the folded SFS for wild, *ex situ*, and commercial seed sources was similar, with few rare alleles, a peak at a frequency around 0.09, and a gradual decline at higher frequencies (Figure 5). The SFS for commercial genotypes could be distinguished from *ex situ* and wild genotypes by a higher abundance of the rarest alleles.

## 4 DISCUSSION

An overarching goal of both restoration and conservation is to maintain evolutionary potential to ensure populations sustain the ability to adapt to change (Hamilton et al., 2020; Hoffmann & Sgro 2011). However, for both *ex situ* conservation collections and seed propagated for restoration, the efficacy of these goals may be dependent upon the amount and type of genetic variation maintained in populations. Sampling effects and genetic bottlenecks associated with seed collection and selection during propagation can create genotypic differences between seed source types. Using a genomic dataset assembled from wild contemporary, commercial, *ex situ*, and selected populations of *H*. *maximiliani*, we tested for the presence of genomic differences that could be attributed to seed source type. We found evidence that commercial seed and selected lines were genetically differentiated from wild and *ex situ* collections. These differences could not be explained by neutral evolutionary processes, such as isolation by distance, implicating other explanations for genomic differences among seed sources. While we did not find direct evidence that selection caused genomic differentiation between seed sources, increased coancestry and low LD‐N_e_ in *ex situ* collections were consistent with an impact of sampling during seed collection. Varying genomic composition of commercial seed sources relative to wild, contemporary populations suggest further study is required to evaluate whether genomic differences correspond to functional differences that impact restoration success. Common garden studies have shown that seed transfer across environments can impact plant traits and performance (Bucharova et al., 2017; Giencke et al., 2018; Johnson et al., 2004; Lesica & Allendorf 1999; Yoko et al., 2020). Consequently, the genomic differences we observe here warrant additional study to link genomic differences in *H*. *maximiliani* seed sources to functional traits and persistence in restored environments. Our work also suggests that similar studies in other plant species are warranted to better understand how restoration seed inputs can evolve and the degree to which genomic differences among seed sources could impact restoration success.

Evolution in commercially produced seed material could be caused by population bottlenecks during initial collection or during cultivated propagation (Espeland et al., 2017; Pizza et al., 2021). A common garden study comparing commercial seed and wild collected seed found fitness was reduced in commercial seed, regardless of whether the environment was stressful or not, consistent with the expectations of a population bottleneck reducing fitness (Pizza et al., 2021). The potential of sampling to affect the genomic composition of collections has also been a concern in *ex situ* conservation. Population bottlenecks can co‐occur with selection caused by the application of fertilizers and pesticides, the use of machinery to harvest seed, or an unnatural biotic environment (Espeland et al., 2017). To our knowledge, only two experimental studies have thus far demonstrated selection in response to agricultural cultivation of restoration seed. The first explicit demonstration that evolution could alter the composition of restoration seed was performed by mixing seed collected from multiple *Elymus glaucus* populations comparing the frequency of electrophoretic markers before and after a single generation of propagation in an agricultural field (Dyer et al., 2016). More recently, Nagel et al. (2019) compared amplified fragment length polymorphisms in five restoration plant species before, during, and after four generations of cultivation. Of the five species, they found that two exhibited significant evidence of genetic differentiation, although only one species also demonstrated a change in traits. Overall, the differences we observed match expectations established by these studies for how evolution via selection and sampling effects could lead to differentiation between commercial and wild populations.

Discriminant analysis of principal components also split the four commercial populations according to the state of their collection, either North or South Dakota. The split among commercial populations was also apparent with PCA, which separated the two populations from North Dakota from all other nonselected populations. Notably, within an individual region, commercial populations were grown by different suppliers despite their genomic similarity. Genetic similarity among different sources of commercial seed could indicate consistent local practices in commercial production or that suppliers are pulling from similar genetic resources. We did not find robust evidence for selection as a cause for the differences between wild and commercial seed, which suggests it is more likely that seed suppliers are using similar genetic stock. Regardless of the reason for this effect, similarity in commercial seed does not match most restoration goals which attempt to balance high genetic diversity with the need for locally adapted seed inputs (Hamilton et al., 2020; Hufford & Mazer 2003; McKay et al., 2005), a problem compounded when commercial seed is not a close analogue to wild populations. Commercial seed is used for restoration because the necessary volume of seed cannot be sustainably harvested from wild populations (Broadhurst et al., 2008). If there are few *H*. *maximiliani* populations of appropriate size for harvesting seed within different regions, it would then be unsurprising if different commercial suppliers obtained and mixed germplasm from the same wild sources. While we do not know the fitness of commercial seed relative to wild genotypes, the genomic dissimilarity between commercial and wild seed warrants greater communication between seed suppliers and restoration practitioners to understand the potential causes of differences observed.

Genomic differences between *H*. *maximiliani* populations were not correlated with geographic distances and do not appear to demonstrate patterns of IBD. In natural populations, genomic differences are expected to increase in response to increasing spatial distance and a corresponding reduction in gene flow among populations (Slatkin 1993; Wright 1943). The absence of IBD in our data could have multiple explanations. First, there could be sufficient gene flow to connect *H*. *maximiliani* populations across the largest spatial scales included in our analysis, but substantial gene flow should also homogenize the genomic variation between populations. This does not correspond to the results of our DAPC analysis, which was able to partition genomic variation, not just at the scale of seed source types, but at the level of individual populations. An alternative cause for the lack of IBD could be rates of gene flow near zero, such that every population is functionally isolated, negating the effect of distance. Although fragmentation of prairie habitat in North America has indeed increased the isolation among plant populations (Samson et al., 2004; Wimberly et al., 2018), the complete cessation of gene flow across populations has not been observed in other species. In the grass *Festuca hallii*, distance was still correlated with genetic variation across the same geographic region considered in our study (Qiu et al., 2009). Although grasses and sunflowers differ in their pollination ecology and methods of seed dispersal, these patterns of differentiation in *F*. *hallii* suggest it is unlikely that prairie plant populations are so isolated that geographic distance has no effect on population structure. Rather, given the structure of our analysis, it is more probable that seed source differences disrupted patterns of IBD and more strongly predicted differences in pairwise F_st_. Increased sampling across commercial and wild populations would be useful to supplement our observations on the effect of seed source type and warrants additional study, particularly given the limited range of *H*. *maximiliani*'s explored by this study. Knowledge of the degree to which commercial propagation disrupts the effects of natural IBD in *H*. *maximiliani* populations and whether similar effects occur in other species would be valuable for restoration practitioners seeking to best match seed inputs to local environmental conditions.

Selection during agricultural propagation can result in the evolution of restoration seed, altering traits that contribute to growth and phenology (Dyer et al., 2016; Nagel et al., 2019). Although commercial populations were genetically distinct from wild contemporary populations, we did not find evidence that differences are due to selection. Commercial and wild populations did not differ in H_e_, F_is_, LD‐N_e_, or coancestry. Tajima's D in commercial populations was also not significantly different from zero, which suggests that selection has not been strong enough to exert genome scale effects. Interestingly, Tajima's D was significantly greater in commercial than wild populations, which should indicate lower frequency of rare alleles in commercial populations. This interpretation contradicts the pattern of allele frequencies seen in our SFS and likely reflects the change in the frequency of rare alleles caused by pooling within seed source types for this analysis. Although selection in agricultural ecosystems is common, experimental cultivation of five different plant species found that molecular evidence of evolution was not apparent in two (Nagel et al., 2019). Species that were perennial or outcrossing, such as *H*. *maximiliani*, were also less likely to exhibit evidence of selection. Thus, although we uncovered multiple ways in which commercial and wild populations differ, the life history and mating system of *H*. *maximiliani* may have buffered against evolutionary change during commercial production. Overall, the genomic differences between commercial and wild populations do not appear to be driven by selection during cultivation, a phenomenon which might be more common in plant species with shorter life histories or that exhibit greater instances of selfing.

We found significant differences in coancestry between *ex situ* and commercial seed sources. *Ex situ* populations also had lower LD‐N_e_ than commercial populations, and although this comparison was not significant, a high coancestry should coincide with higher rates of linkage disequilibrium and lower LD‐N_e_. Low LD‐N_e_ and higher coancestry without corresponding increases in F_is_ could reflect the sampling methods used to establish these collections. Alleles are more likely to be identical by descent in populations with greater coancestry and are less likely to represent the uniform sampling of large populations (Cavalli‐Sforza & Bodmer 1971). In *ex situ* seed collections, high coancestry and low LD‐N_e_ could result from sampling large quantities of seed from a relatively small number of maternal individuals. Sampling in this manner would also not immediately reduce H_e_ or increase F_is_ in a self‐incompatible species prior to sexual reproduction (Allendorf 1986; Leberg 1992), but would increase coancestry and LD‐N_e_ because of the large number of half‐siblings represented in the collection. The difference between commercial and *ex situ* collections may imply that commercial seed provides a superior resource by harboring greater genotypic diversity. Whether or not this is true likely depends on the specific goal of the collection. For example, high coancestry could be mitigated if multiple *ex situ* collections are mated prior to deployment in the wild. Additionally, genomic clustering analyses indicate *ex situ* collections are closer analogues to contemporary wild populations and could be superior resources for restoration if the genotypic differences depicted in our analysis correlate with functional differences. This suggests the need for additional work to evaluate the consequences of high coancestry and genomic differences from wild populations and will be essential for applying our results into practice for restoration.

The production of seed for restoration and conservation includes an inherent conflict between maintaining the genomic composition of wild populations and supplying large volumes of seed (Broadhurst et al., 2008; Espeland et al., 2017). In addition to these challenges, the goals of conservation are themselves sometimes in conflict, with the need to maintain populations that are locally adapted while maximizing genetic diversity to buffer against contemporary and future environmental challenges, respectively (Bucharova et al., 2017; Hamilton et al., 2020). The loss of genetic diversity and evolution of functional traits during cultivation is thus a major concern for restoration efforts. In our comparison of commercial and wild *H*. *maximiliani* collections, we did not find evidence of selection or reduced genetic variation in commercial seed, but we did observe significant differences in their genotypic composition. Additionally, the surprising genomic similarity of commercial seed sourced from the same region is evidence for a homogenizing factor either during seed collection or cultivation. High similarity across commercial seed inputs is at odds with the goal of maximizing genetic diversity while maintaining local adaptation and has the potential to reduce the efficacy of restoration in the short and long term (McKay et al., 2005). Given the species‐specific evolutionary consequences of cultivation (Nagel et al., 2019), it is also possible that other seed inputs which are less buffered against the genomic effects of selection, due to their life history or mating strategies, will exhibit increased differentiation from wild populations during commercial production (Ballesteros‐Mejia et al., 2016; Hamrick et al., 1979). Additional study evaluating the trait variation and contribution of *H*. *maximiliani* to ecosystem services between wild and commercial seed collected across varied restored habitats is necessary. Furthermore, to fully integrate the consequences of our study for restoration, similar work comparing plant species commonly used in restoration will be important for generalizing these results. Until this work can be performed, increased collaboration between producers and users of commercial seed is needed to better understand the effects of provenance, individual methods of harvest, and cultivation on seed material needed to best meet restoration goals (Hamilton et al., 2020).

## CONFLICT OF INTEREST

None declared.

## Supporting information

Supplementary Material

## Data Availability

Raw sequence data associated with this project have been archived and are openly available at the National Center for Biotechnology Information short read sequence archive at https://www.ncbi.nlm.nih.gov/sra under BioProject PRJNA735802. Other data and code associated with the project have been made available on GitHub at https://github.com/Joebraasch/Helianthus_maximiliani_project.

## References

[eva13275-bib-0001] Allendorf, F. W. (1986). Genetic drift and the loss of alleles versus heterozygosity. Zoo Biology, 5(2), 181–190. 10.1002/zoo.1430050212

[eva13275-bib-0002] Angeloni, F., Ouborg, N. J., & Leimu, R. (2011). Meta‐analysis on the association of population size and life history with inbreeding depression in plants. Biological Conservation, 144(1), 35–43. 10.1016/j.biocon.2010.08.016

[eva13275-bib-0003] Archer, F. I., Adams, P. E., & Schneiders, B. B. (2017). stratag: An r package for manipulating, summarizing and analysing population genetic data. Molecular Ecology Resources, 17(1), 5–11. 27327208 10.1111/1755-0998.12559

[eva13275-bib-0004] Asselin, S. R., Brûlé‐Babel, A. L., Van Tassel, D. L., & Cattani, D. J. (2020). Genetic analysis of domestication parallels in annual and perennial sunflowers (Helianthus spp.): routes to crop development. Frontiers in Plant Science, 11, 834. 32595690 10.3389/fpls.2020.00834 PMC7304338

[eva13275-bib-0005] Ballesteros‐Mejia, L., Lima, N. E., Lima Ribeiro, M. S., & Collevatti, R. G. (2016). Pollination mode and mating system explain patterns in genetic differentiation in neotropical plants. PLoS One, 11(7), e0158660. 10.1371/journal.pone.0158660 27472384 PMC4966973

[eva13275-bib-0006] Benayas, J. M., Newton, A. C., Diaz, A., & Bullock, J. M. (2009). Enhancement of biodiversity and ecosystem services by ecological restoration: a meta‐analysis. Science, 325(5944), 1121–1124. 19644076 10.1126/science.1172460

[eva13275-bib-0007] Bischoff, A., Vonlanthen, B., Steinger, T., & Müller‐Schärer, H. (2006). Seed provenance matters—effects on germination of four plant species used for ecological restoration. Basic and Applied Ecology, 7(4), 347–359. 10.1016/j.baae.2005.07.009

[eva13275-bib-0008] Blanquart, F., Kaltz, O., Nuismer, S. L., & Gandon, S. (2013). A practical guide to measuring local adaptation. Ecology Letters, 16(9), 1195–1205. 10.1111/ele.12150 23848550

[eva13275-bib-0009] Bolger, A. M., Lohse, M., & Usadel, B. (2014). Trimmomatic: a flexible trimmer for Illumina sequence data. Bioinformatics, 30(15), 2114–2120. 10.1093/bioinformatics/btu170 24695404 PMC4103590

[eva13275-bib-0010] Braasch, J., Barker, B. S., & Dlugosch, K. M. (2019). Expansion history and environmental suitability shape effective population size in a plant invasion. Molecular Ecology, 28(10), 2546–2558. 10.1111/mec.15104 30993767 PMC6584048

[eva13275-bib-0011] Bradburd, G. S., Ralph, P. L., & Coop, G. M. (2016). A spatial framework for understanding population structure and admixture. PLoS Genetics, 12(1), e1005703. 10.1371/journal.pgen.1005703 26771578 PMC4714911

[eva13275-bib-0012] Broadhurst, L. M., Lowe, A., Coates, D. J., Cunningham, S. A., McDonald, M., Vesk, P. A., & Yates, C. (2008). Seed supply for broadscale restoration: maximizing evolutionary potential. Evolutionary Applications, 1(4), 587–597. 10.1111/j.1752-4571.2008.00045.x 25567799 PMC3352390

[eva13275-bib-0013] Bucharova, A., Durka, W., Hölzel, N., Kollmann, J., Michalski, S., & Bossdorf, O. (2017). Are local plants the best for ecosystem restoration? It depends on how you analyze the data. Ecology and Evolution, 7(24), 10683–10689. 10.1002/ece3.3585 29299248 PMC5743477

[eva13275-bib-0014] Caballero, A., & Toro, M. A. (2000). Interrelations between effective population size and other pedigree tools for the management of conserved populations. Genetical Research, 75(3), 331–343. 10893869 10.1017/s0016672399004449

[eva13275-bib-0015] Cavalli‐Sforza, L. L., & Bodmer, W. F. (1971). The Genetics of Human Populations. W. H. Freeman and Company.

[eva13275-bib-0016] Comer, P. J., Hak, J. C., Kindscher, K., Muldavin, E., & Singhurst, J. (2018). Continent‐scale landscape conservation design for temperate grasslands of the Great Plains and Chihuahuan Desert. Natural Areas Journal, 38(2), 196–211. 10.3375/043.038.0209

[eva13275-bib-0017] DiSanto, L. N., & Hamilton, J. A. (2020). Using environmental and geographic data to optimize ex situ collections and preserve evolutionary potential. Conservation Biology: the Journal of the Society for Conservation Biology, 7, 12. 10.1111/cobi.13568 32519757

[eva13275-bib-0018] Diwan, N., McIntosh, M. S., & Bauchan, G. R. (1995). Methods of developing a core collection of annual Medicago species. TAG. Theoretical and Applied Genetics. Theoretische Und Angewandte Genetik, 90(6), 755–761. 10.1007/BF00222008 24172915

[eva13275-bib-0019] Dyer, A. R., Knapp, E. E., & Rice, K. J. (2016). Unintentional selection and genetic changes in native perennial grass populations during commercial seed production. Ecological Restoration, 34(1), 39–48. 10.3368/er.34.1.39

[eva13275-bib-0020] Eaton, D. A. R. (2014). PyRAD: assembly of de novo RADseq loci for phylogenetic analyses. Bioinformatics, 30(13), 1844–1849. 10.1093/bioinformatics/btu121 24603985

[eva13275-bib-0021] Elshire, R. J., Glaubitz, J. C., Sun, Q., Poland, J. A., Kawamoto, K., Buckler, E. S., & Mitchell, S. E. (2011). A robust, simple genotyping‐by‐sequencing (GBS) approach for high diversity species. PLoS One, 6(5), e19379. 10.1371/journal.pone.0019379 21573248 PMC3087801

[eva13275-bib-0022] Espeland, E. K., Emery, N. C., Mercer, K. L., Woolbright, S. A., Kettenring, K. M., Gepts, P., & Etterson, J. R. (2017). Evolution of plant materials for ecological restoration: insights from the applied and basic literature. The Journal of Applied Ecology, 54(1), 102–115. 10.1111/1365-2664.12739

[eva13275-bib-0023] Excoffier, L., Foll, M., & Petit, R. J. (2009). Genetic consequences of range expansions. Annual Review of Ecology, Evolution, and Systematics, 40(1), 481–501. 10.1146/annurev.ecolsys.39.110707.173414

[eva13275-bib-0024] Fant, J. B., Holmstrom, R. M., Sirkin, E., Etterson, J. R., & Masi, S. (2008). Genetic structure of threatened native populations and propagules used for restoration in a clonal species, American beachgrass (Ammophila breviligulata Fern.). Restoration Ecology, 16(4), 594–603.

[eva13275-bib-0025] Franco, J., Crossa, J., Taba, S., & Shands, H. (2005). A sampling strategy for conserving genetic diversity when forming core subsets. Crop Science, 45(3), 1035–1044. 10.2135/cropsci2004.0292

[eva13275-bib-0026] García‐Cortés, L. A., Martínez‐Ávila, J. C., & Toro, M. A. (2010). Fine decomposition of the inbreeding and the coancestry coefficients by using the tabular method. Conservation Genetics, 11, 1945–1952. 10.1007/s10592-010-0084-x

[eva13275-bib-0027] Garrison, E., & Marth, G. (2012). Haplotype‐based variant detection from short‐read sequencing. In *arXiv [q‐bio.GN]*. arXiv. http://arxiv.org/abs/1207.3907

[eva13275-bib-0097] Gerla, P. J., Cornett, M. W., Ekstein, J. D., & Ahlering, M. A. (2012). Talking big: Lessons learned from a 9000 hectare restoration in the Northern Tallgrass Prairie. Sustainability, 4(11), 3066–3087. 10.3390/su4113066

[eva13275-bib-0028] Giencke, L. M., & Carol Denhof, R. (2018). Seed sourcing for longleaf pine ground cover restoration: using plant performance to assess seed transfer zones and home‐site advantage. Restoration, 26(6), 1127–1136. 10.1111/rec.12673

[eva13275-bib-0029] Griffith, M. P., Calonje, M., Meerow, A. W., Tut, F., Kramer, A. T., Hird, A., Magellan, T. M., & Husby, C. E. (2015). Can a botanic garden cycad collection capture the genetic diversity in a wild population? The International Journal of Plant Science, 176(1), 1–10. 10.1086/678466

[eva13275-bib-0030] Griffith, M. P., Cartwright, F., Dosmann, M., Fant, J., Freid, E., Havens, K., Jestrow, B., Kramer, A. T., Magellan, T. M., Meerow, A. W., Meyer, A., Sanchez, V., Santiago‐Valentín, E., Spence, E., Sustasche‐Sustache, J. A., Francisco‐Ortega, J., & Hoban, S. (2021). Ex situ conservation of large and small plant populations illustrates limitations of common conservation metrics. The International Journal of Plant Science, 182(4), 263–276. 10.1086/713446

[eva13275-bib-0031] Hamilton, J., Flint, S., Lindstrom, J., Volk, K., Shaw, R., & Ahlering, M. (2020). Evolutionary approaches to seed sourcing for grassland restorations. The New Phytologist, 225(6), 2246–2248. 10.1111/nph.16427 32064631

[eva13275-bib-0032] Hamilton, M. B. (1994). Ex situ conservation of wild plant species: time to reassess the genetic assumptions and implications of seed banks. Conservation Biology, 8(1), 39–49. 10.1046/j.1523-1739.1994.08010039.x

[eva13275-bib-0033] Hamrick, J. L., Linhart, Y. B., & Mitton, J. B. (1979). Relationships between life history characteristics and electrophoretically detectable genetic variation in plants. Annual Review of Ecology and Systematics, 10, 173–200. 10.1146/annurev.es.10.110179.001133

[eva13275-bib-0034] Hoban, S., & Schlarbaum, S. (2014). Optimal sampling of seeds from plant populations for *ex‐situ* conservation of genetic biodiversity, considering realistic population structure. Biological Conservation, 177, 90–99. 10.1016/j.biocon.2014.06.014

[eva13275-bib-0035] Hobbs, R. J., & Norton, D. A. (1996). Towards a conceptual framework for restoration ecology. Restoration Ecology, 4(2), 93–110. 10.1111/j.1526-100X.1996.tb00112.x

[eva13275-bib-0036] Hodgson, J. A., Wallis, D. W., Krishna, R., & Cornell, S. J. (2016). How to manipulate landscapes to improve the potential for range expansion. Methods in Ecology and Evolution/British Ecological Society, 7(12), 1558–1566. 10.1111/2041-210X.12614

[eva13275-bib-0037] Hoekstra, J. M., Boucher, T. M., Ricketts, T. H., & Roberts, C. (2004). Confronting a biome crisis: global disparities of habitat loss and protection. Ecology Letters, 8(1), 23–29. 10.1111/j.1461-0248.2004.00686.x

[eva13275-bib-0038] Hoffmann, A. A., & Sgrò, C. M. (2011). Climate change and evolutionary adaptation. Nature, 470(7335), 479–485. 21350480 10.1038/nature09670

[eva13275-bib-0039] Hohenlohe, P. A., Phillips, P. C., & Cresko, W. A. (2010). Using population genomics to detect selection in natural populations: key concepts and methodological considerations. International Journal of Plant Sciences, 171(9), 1059–1071. 21218185 10.1086/656306 PMC3016716

[eva13275-bib-0040] Hufford, K. M., & Mazer, S. J. (2003). Plant ecotypes: genetic differentiation in the age of ecological restoration. Trends in Ecology & Evolution, 18(3), 147–155. 10.1016/S0169-5347(03)00002-8

[eva13275-bib-0041] Hughes, A. R., Inouye, B. D., Johnson, M. T. J., Underwood, N., & Vellend, M. (2008). Ecological consequences of genetic diversity. Ecology Letters, 11(6), 609–623. 10.1111/j.1461-0248.2008.01179.x 18400018

[eva13275-bib-0042] Husband, B. C., & Schemske, D. W. (1996). Evolution of the magnitude and timing of inbreeding depression in plants. Evolution; International Journal of Organic Evolution, 50(1), 54–70. 10.1111/j.1558-5646.1996.tb04472.x 28568860

[eva13275-bib-0043] Johnson, G. R., Sorensen, F. C., St Clair, J. B., & Cronn, R. C. (2004). Pacific Northwest Forest Tree Seed Zones: A template for native plants? Native Plants Journal, 5(2), 131–140. 10.2979/NPJ.2004.5.2.131

[eva13275-bib-0044] Jombart, T. (2008). adegenet: A R package for the multivariate analysis of genetic markers. Bioinformatics, 24(11), 1403–1405. 10.1093/bioinformatics/btn129 18397895

[eva13275-bib-0045] Jombart, T., & Ahmed, I. (2011). adegenet 1.3‐1: new tools for the analysis of genome‐wide SNP data. Bioinformatics, 27(21), 3070–3071. 21926124 10.1093/bioinformatics/btr521 PMC3198581

[eva13275-bib-0046] Jombart, T., Devillard, S., & Balloux, F. (2010). Discriminant analysis of principal components: a new method for the analysis of genetically structured populations. BMC Genetics, 11, 94. 10.1186/1471-2156-11-94 20950446 PMC2973851

[eva13275-bib-0047] Kawakami, T., Darby, B. J., & Ungerer, M. C. (2014). Transcriptome resources for the perennial sunflower Helianthus maximiliani obtained from ecologically divergent populations. Molecular Ecology Resources, 14(4), 812–819. 24438509 10.1111/1755-0998.12227

[eva13275-bib-0048] Kawecki, T. J., & Ebert, D. (2004). Conceptual issues in local adaptation. Ecology Letters, 7(12), 1225–1241. 10.1111/j.1461-0248.2004.00684.x

[eva13275-bib-0049] Keller, L. F., & Waller, D. M. (2002). Inbreeding effects in wild populations. Trends in Ecology & Evolution, 17(5), 230–241. 10.1016/S0169-5347(02)02489-8

[eva13275-bib-0050] Keller, M., Kollmann, J., & Edwards, P. J. (2000). Genetic introgression from distant provenances reduces fitness in local weed populations. The Journal of Applied Ecology, 37(4), 647–659. 10.1046/j.1365-2664.2000.00517.x

[eva13275-bib-0051] Kimball, S., Lulow, M., Sorenson, Q., Balazs, K., Fang, Y.‐C., Davis, S. J., O’Connell, M., & Huxman, T. E. (2015). Cost‐effective ecological restoration. Restoration Ecology, 23(6), 800–810. 10.1111/rec.12261

[eva13275-bib-0052] Kuznetsova, A., Brockhoff, P. B., Christensen, R. H. B., & Others. (2017). lmerTest package: tests in linear mixed effects models. Journal of Statistical Software, 82(13), 1–26.

[eva13275-bib-0053] Leberg, P. L. (1992). Effects of population bottlenecks on genetic diversity as measured by allozyme electrophoresis. Evolution; International Journal of Organic Evolution, 46(2), 477–494. 10.1111/j.1558-5646.1992.tb02053.x 28564024

[eva13275-bib-0054] Lesica, P., & Allendorf, F. W. (1999). Ecological genetics and the restoration of plant communities: Mix or match? Restoration Ecology, 7(1), 42–50. 10.1046/j.1526-100X.1999.07105.x

[eva13275-bib-0096] Li, H. (2014). Toward better understanding of artifacts in variant calling from high‐coverage samples. Bioinformatics, 30(20), 2843–2851. 24974202 10.1093/bioinformatics/btu356 PMC4271055

[eva13275-bib-0055] Li, D.‐Z., & Pritchard, H. W. (2009). The science and economics of ex situ plant conservation. Trends in Plant Science, 14(11), 614–621. 10.1016/j.tplants.2009.09.005 19818672

[eva13275-bib-0056] Li, H., & Durbin, R. (2009). Fast and accurate short read alignment with Burrows‐Wheeler transform. Bioinformatics, 25(14), 1754–1760. 10.1093/bioinformatics/btp324 19451168 PMC2705234

[eva13275-bib-0057] Maruyama, T., & Fuerst, P. A. (1985). Population bottlenecks and nonequilibrium models in population genetics. II. Number of alleles in a small population that was formed by a recent bottleneck. Genetics, 111(3), 675–689. 4054612 10.1093/genetics/111.3.675 PMC1202664

[eva13275-bib-0058] McKay, J. K., Christian, C. E., Harrison, S., & Rice, K. J. (2005). “How local is local?”—a review of practical and conceptual issues in the genetics of restoration. Restoration Ecology, 13(3), 432–440. 10.1111/j.1526-100X.2005.00058.x

[eva13275-bib-0059] McKenna, T. P., McDonnell, J., Yurkonis, K. A., & Brophy, C. (2019). *Helianthus maximiliani* and species fine‐scale spatial pattern affect diversity interactions in reconstructed tallgrass prairies. Ecology and Evolution, 9(21), 12171–12181. 31832151 10.1002/ece3.5696 PMC6854329

[eva13275-bib-0060] Merritt, D. J., & Dixon, K. W. (2011). Restoration seed banks—a matter of scale. Science, 332(6028), 424–425. 21512021 10.1126/science.1203083

[eva13275-bib-0061] Nagel, R., Durka, W., Bossdorf, O., & Bucharova, A. (2019). Rapid evolution in native plants cultivated for ecological restoration: not a general pattern. Plant Biology, 21(3), 551–558. 10.1111/plb.12901 30120869

[eva13275-bib-0062] Narum, S. R., & Hess, J. E. (2011). Comparison of FST outlier tests for SNP loci under selection. Molecular Ecology Resources, 11(Suppl. 1), 184–194. 21429174 10.1111/j.1755-0998.2011.02987.x

[eva13275-bib-0063] Nielsen, R. (2001). Statistical tests of selective neutrality in the age of genomics. Heredity, 86(Pt 6), 641–647. 10.1046/j.1365-2540.2001.00895.x 11595044

[eva13275-bib-0064] Paradis, E. (2010). pegas: An R package for population genetics with an integrated–modular approach. Bioinformatics, 26(3), 419–420. 10.1093/bioinformatics/btp696 20080509

[eva13275-bib-0065] Pedrini, S., Gibson‐Roy, P., Trivedi, C., Gálvez‐Ramírez, C., Hardwick, K., Shaw, N., Frischie, S., Laverack, G., & Dixon, K. (2020). Collection and production of native seeds for ecological restoration. Restoration Ecology, 28(S3), 198.

[eva13275-bib-0066] Pizza, R., Espleand, E., & Etterson, J. (2021). Eight generations of native seed cultivation reduces plant fitness relative to the wild progenitor population. Evolutionary Applications. 10.1111/eva.13243 PMC8288025 34295366

[eva13275-bib-0067] Puritz, J. B., Hollenbeck, C. M., & Gold, J. R. (2014). dDocent: a RADseq, variant‐calling pipeline designed for population genomics of non‐model organisms. PeerJ, 2, e431. 24949246 10.7717/peerj.431 PMC4060032

[eva13275-bib-0068] Puritz, J. B., Matz, M. V., Toonen, R. J., Weber, J. N., Bolnick, D. I., & Bird, C. E. (2014). Demystifying the RAD fad. Molecular Ecology, 23(24), 5937–5942. 25319241 10.1111/mec.12965

[eva13275-bib-0069] Qiu, J., Fu, Y.‐B., Bai, Y., & Wilmshurst, J. F. (2009). Genetic variation in remnant *Festuca hallii* populations is weakly differentiated, but geographically associated across the Canadian Prairie. Plant Species Biology, 24(3), 156–168.

[eva13275-bib-0070] Robichaux, R. H., Friar, E. A., & Mount, D. W. (1997). Molecular genetic consequences of a population bottleneck associated with reintroduction of the Mauna Kea Silversword (*Argyroxiphium sandwicense* ssp. sandwicense [Asteraceae]). Conservation Biology: the Journal of the Society for Conservation Biology, 11(5), 1140–1146.

[eva13275-bib-0071] Robusto, C. C. (1957). The cosine‐haversine formula. The American Mathematical Monthly, 64(1), 38–40. 10.2307/2309088

[eva13275-bib-0072] Roesti, M., Salzburger, W., & Berner, D. (2012). Uninformative polymorphisms bias genome scans for signatures of selection. BMC Evolutionary Biology, 12, 94. 10.1186/1471-2148-12-94 22726891 PMC3426483

[eva13275-bib-0073] Roundy, B. A., Shaw, N. L., & Booth, D. T. (1997) Using native seeds on rangelands. In N.L. Shaw and B.A. Roundy (comps.) Proceedings: Using seeds of native species on rangelands. Gen. Tech. Rep. INT‐GTR‐372. U.S. Department of Agriculture, Forest Service, Intermountain Research Station. (pp. 1–8). Ogden, UT, USA.

[eva13275-bib-0074] Samson, F. B., Knopf, F. L., & Ostlie, W. R. (2004). Great Plains ecosystems: past, present, and future. Wildlife Society Bulletin, 32(1), 6–15.

[eva13275-bib-0075] Schoen, D. J., & Brown, A. H. (1993). Conservation of allelic richness in wild crop relatives is aided by assessment of genetic markers. Proceedings of the National Academy of Sciences of the United States of America, 90(22), 10623–10627. 10.1073/pnas.90.22.10623 8248153 PMC47829

[eva13275-bib-0076] Seiler, G. J. (2010). Germination and viability of wild sunflower species achenes stored at room temperature for 20 years. Seed Science and Technology, 38(3), 786–791. 10.15258/sst.2010.38.3.27

[eva13275-bib-0077] Siberchicot, A., Julien‐Laferrière, A., Dufour, A.‐B., Thioulouse, J., & Dray, S. (2017). adegraphics: an S4 lattice‐based package for the representation of multivariate data. The R Journal, 9(2), 198. https://journal.r‐project.org/archive/2017/RJ‐2017‐042/RJ‐2017‐042.pdf. 10.32614/RJ-2017-042

[eva13275-bib-0078] Simonsen, K. L., Churchill, G. A., & Aquadro, C. F. (1995). Properties of statistical tests of neutrality for DNA polymorphism data. Genetics, 141(1), 413–429. 10.1093/genetics/141.1.413 8536987 PMC1206737

[eva13275-bib-0079] Slatkin, M. (1993). Isolation by distance in equilibrium and non‐equilibrium populations. Evolution, 47(1), 264–279. 10.1111/j.1558-5646.1993.tb01215.x 28568097

[eva13275-bib-0080] Taft, H. R., McCoskey, D. N., Miller, J. M., Pearson, S. K., Coleman, M. A., Fletcher, N. K., Mittan, C. S., Meek, M. H., & Barbosa, S. (2020). Research–management partnerships: An opportunity to integrate genetics in conservation actions. Conservation Science and Practice, 2(9), 1–8. 10.1111/csp2.218

[eva13275-bib-0081] Tajima, F. (1983). Evolutionary relationship of DNA sequences in finite populations. Genetics, 105(2), 437–460. 10.1093/genetics/105.2.437 6628982 PMC1202167

[eva13275-bib-0082] Tajima, F. (1989). The effect of change in population size on DNA polymorphism. Genetics, 123(3), 597–601. 10.1093/genetics/123.3.597 2599369 PMC1203832

[eva13275-bib-0083] Tetreault, H. M., Kawakami, T., & Ungerer, M. C. (2016). Low temperature tolerance in the perennial sunflower *Helianthus maximiliani* . The American Midland Naturalist, 175(1), 91–102. 10.1674/amid-175-01-91-102.1

[eva13275-bib-0084] Thomson, J. R., Moilanen, A. J., Vesk, P. A., Bennett, A. F., & Nally, R. M. (2009). Where and when to revegetate: a quantitative method for scheduling landscape reconstruction. Ecological Applications: A Publication of the Ecological Society of America, 19(4), 817–828. 10.1890/08-0915.1 19544726

[eva13275-bib-0085] United States Department of Agriculture (2004). USDA NRCS Plant Guide; Maximilian Sunflower. https://plants.sc.egov.usda.gov/plantguide/pdf/pg_hema2.pdf

[eva13275-bib-0086] Wang, J. (2011). COANCESTRY: a program for simulating, estimating and analyzing relatedness and inbreeding coefficients. Molecular Ecology Resources, 11(1), 141–145. 21429111 10.1111/j.1755-0998.2010.02885.x

[eva13275-bib-0087] Waples, R. K., Larson, W. A., & Waples, R. S. (2016). Estimating contemporary effective population size in non‐model species using linkage disequilibrium across thousands of loci. Heredity, 117, 233–240. 10.1038/hdy.2016.60 27553452 PMC5026758

[eva13275-bib-0088] Weir, B. S., & Cockerham, C. C. (1984). Estimating f‐statistics for the analysis of population structure. Evolution, 38(6), 1358‐1370. 28563791 10.1111/j.1558-5646.1984.tb05657.x

[eva13275-bib-0089] Williams, S. L. (2001). Reduced genetic diversity in eelgrass transplantations affects both population growth and individual fitness. In M. D. Bertness, S. D. Gaines, & M. E. Hay (Eds.), Marine Community Ecology (pp. 317–337). Sinauer Associates.

[eva13275-bib-0090] Willing, E.‐M., Dreyer, C., & van Oosterhout, C. (2012). Estimates of genetic differentiation measured by F(ST) do not necessarily require large sample sizes when using many SNP markers. PLoS One, 7(8), e42649. 10.1371/journal.pone.0042649 22905157 PMC3419229

[eva13275-bib-0091] Wimberly, M. C., Narem, D. M., Bauman, P. J., Carlson, B. T., & Ahlering, M. A. (2018). Grassland connectivity in fragmented agricultural landscapes of the north‐central United States. Biological Conservation, 217, 121–130. 10.1016/j.biocon.2017.10.031

[eva13275-bib-0092] Wright, S. (1938). Size of population and breeding structure in relation to evolution. Science, 87(2263), 430–431.

[eva13275-bib-0093] Wright, S. (1943). Isolation by distance. Genetics, 28(2), 114–138. 10.1093/genetics/28.2.114 17247074 PMC1209196

[eva13275-bib-0094] Yoko, Z. G., Volk, K. L., Dochtermann, N. A., & Hamilton, J. A. (2020). The importance of quantitative trait differentiation in restoration: landscape heterogeneity and functional traits inform seed transfer guidelines. AoB Plants, 12(2), laa009. 10.1093/aobpla/plaa009 PMC7112727 32257091

[eva13275-bib-0095] Zeileis, A., & Hothorn, T. (2002). Diagnostic checking in regression relationships. pkg.cs.ovgu.de. http://pkg.cs.ovgu.de/LNF/i386/5.10/R/LNFr‐lmtest/reloc/R‐2.10/library/lmtest/doc/lmtest‐intro.pdf

