# Supplementary material for: Testing for evolutionary change in restoration: A genomic comparison between ex situ, native, and commercial seed sources of Helianthus maximiliani
Source: Evol Appl. 2021 Jul 19;14(9):2206–20. doi: 10.1111/eva.13275 (PMC8477598; doi:10.1111/eva.13275)
Supplement: Supplementary file 1 — Supplementary Material [file EVA-14-2206-s001.docx]

Table S1. Tajima’s D values for all populations. P-vales indicate whether Tajima’s D values deviate from neutral expectations.


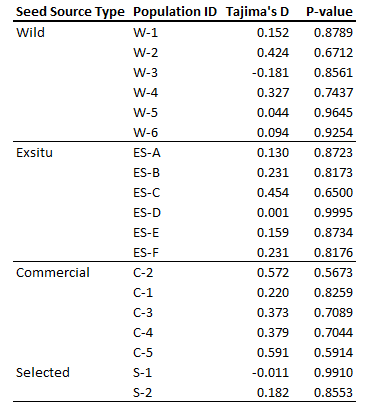


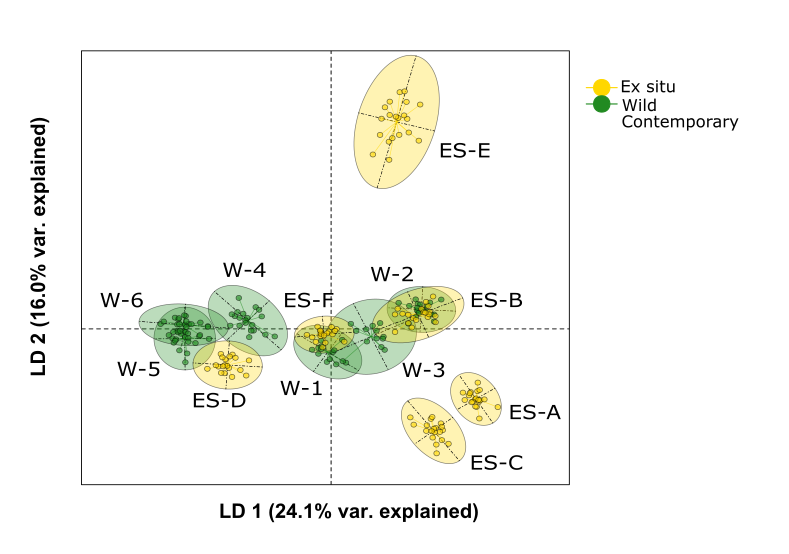


Figure S1. Discriminant analysis of principal components for *Helianthus maximiliani* SNP genotypes from *ex situ* and wild contemporary populations only. Different seed source types are depicted as different colors (yellow: *ex situ*; green: wild contemporary).


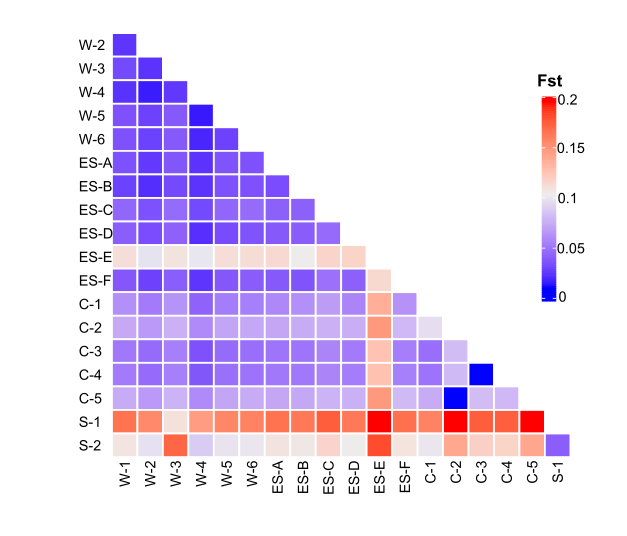


Figure S2. Heatmap of pairwise F_st_ for 19 populations of *H. maximiliani*. F_st_ was calculated using the Wier and Cockerham method.


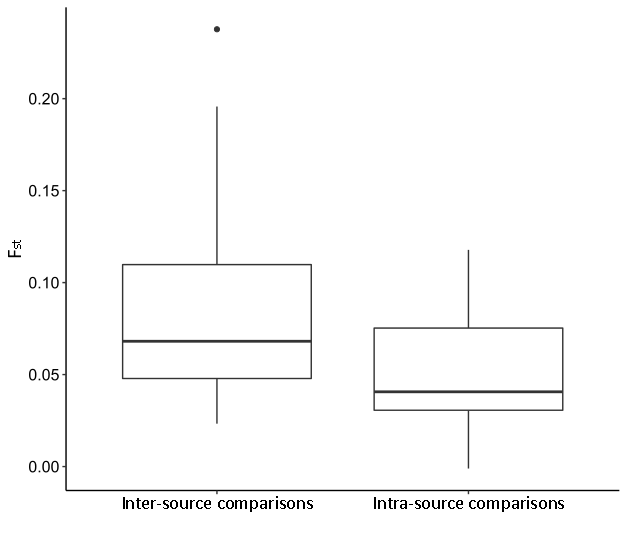


Figure S3. Comparisons between pairwise-Fst calculated for intra-seed source comparisons (both populations are the same seed source type) are lower than inter-population comparisons (the two populations are different seed source types) (P < 0.001).
